# Case of Prolapsus Vesicæ

**Published:** 1851-10

**Authors:** G. D. Crosthwait

**Affiliations:** Iowa City, Iowa


*Case of Prolapsus Vesicæ.* By G. D. Crosthwait, M. D., of Iowa City, Iowa.—Mrs. ———, aged 33 years, of nervo-sanguineous temperament, and delicate general health, and the mother of seven children, consulted me in regard to what she supposed to be prolapsus uteri, and from her description of symptoms, what I also supposed to be that affection. She had frequent pain in her back, pain on the front of her thighs, sense of dragging and bearing down, and in fact no symptom seemed to be wanting to constitute prolapsus uteri of a character so grave as on many occasions to amount to procidentia. In order to determine the degree of the disease and the condition of the parts, I proposed an examination, which was assented to. I was somewhat surprised to find the uterus in its natural relation, or at least as nearly so as is usual in women of her diathesis and circumstances, and was compelled to suggest that she must be mistaken in reference to the procidentia, or at least its extent. She declared that it was so plain and palpable that there could be no mistake about it, but that it was easily returned, and often receded spontaneously. Under these circumstances, I could do no more than to request her, when the tumor again made its appearance, to let it remain until I could be notified and examine it. Within a few days I received the notification, and proceeded to the examination. I found a tumor as large as a hen's egg, or larger, proceeding from immediately behind the symphisis pubis, protruding considerably out of the vulva. There was no difficulty in determining that the protruding body was not the uterus, and on passing the finger beyond the tumor, that organ was found in its proper relation. The tumor was easily returned, though not without pain. This condition of things struck me with some surprise, but there was no difficuly in determining that the affection was a prolapsus, or hernia of the bladder. Its spontaneous reduction being owing generally, if not always, to the emptying of that viscus.

If, however, the diagnosis of the affection under examination at a suitable time became easy, it was far otherwise with regard to the remedy, if there be in fact a remedy which will cure it. I advised her to be prudent in diet and exercise, to avoid constipation, and to attend frequently and very promptly upon all calls to the discharge of urine. This course, indeed, renders the patient's situation very tolerably comfortable, but if the hernia takes place at any time when she is walking, or from home, under circumstances which prevent its manual reduction, she suffers very severe pain.

No pessary or other mechanical contrivance has been advised so far, for the reason that none, the construction of which I am familiar with, would seem to promise much, if any good. In fact, I have feared to recommend an ordinary pessary, lest its position being above the aperture through which the hernia makes its exit might, by its downward pressure, tend to strangulate the hernia, or excite vesical inflammation.

I have not been able, in the limited access to books which my present residence affords, to find any case recorded which at all resembles the one under consideration. I therefore request the consideration of the subject by the society, with such suggestions on the treatment as the wisdom of its members may devise.

The history of the case shows that the affection has existed for several years.—*Ibid.*

